# Revisiting Therapeutic Strategies for *H. pylori* Treatment in the Context of Antibiotic Resistance: Focus on Alternative and Complementary Therapies

**DOI:** 10.3390/molecules26196078

**Published:** 2021-10-08

**Authors:** Ioana Alexandra Cardos, Dana Carmen Zaha, Rakesh K. Sindhu, Simona Cavalu

**Affiliations:** 1 Faculty of Medicine and Pharmacy, Doctoral School of Biomedical Sciences, University of Oradea, 1 University Street, 410087 Oradea, Romania; pfidrcardosioana@gmail.com; 2 Department of Preclinical Sciences, Faculty of Medicine and Pharmacy, University of Oradea, 1 University Street, 410087 Oradea, Romania; 3 Chitkara College of Pharmacy, Chitkara University, Chandigarh 140401, India

**Keywords:** *Helicobacter pylori*, antibiotic resistance, alternative therapy, nanoformulations

## Abstract

The prevalence of *Helicobacter pylori* infection remains significant worldwide and it depends on many factors: gender, age, socio-economic status, geographic area, diet, and lifestyle. All successful infectious diseases treatments use antibiotic-susceptibility testing, but this strategy is not currently practical for *H. pylori* and the usual cure rates of *H. pylori* are lower than other bacterial infections. Actually, there is no treatment that ensures complete eradication of this pathogen. In the context of an alarming increase in resistance to antibiotics (especially to clarithromycin and metronidazole), alternative and complementary options and strategies are taken into consideration. As the success of antibacterial therapy depends not only on the susceptibility to given drugs, but also on the specific doses, formulations, use of adjuvants, treatment duration, and reinfection rates, this review discusses the current therapies for *H. pylori* treatment along with their advantages and limitations. As an alternative option, this work offers an extensively referenced approach on natural medicines against *H. pylori*, including the significance of nanotechnology in developing new strategies for treatment of *H. pylori* infection.

## 1. Introduction

The *Helicobacter pylori* is a helix-shaped, microaerophilic, Gram-negative bacterium, with high motility owing to its four to six flagella, capable of colonizing the gastric mucosa and thereby establishing infection. Most infected subjects remain asymptomatic for a long time, but this colonization produces a silent destruction of the gastric mucosa. Since its discovery in 1982, by Australian doctors Barry Marshall and Robin Warren, more and more studies have proven that *H. pylori* is responsible for atrophic gastritis, peptic ulcer, and gastric malignancies [1,2]. From a physiological point of view, *H. pylori* is microaerophilic, requiring oxygen to survive, but at a lower concentration than in the atmosphere. Another special feature of this bacteria is its nitrogen metabolism. As amino acids and urea are well known major sources of nitrogen in the gastric environment, the main necessary amino acids for *H. pylori* are valine, methionine, arginine, histidine, isoleucine, leucine, phenylalanine, fermentation being the most important mode of amino acid utilization [3]. On the other hand, all *H. pylori* strains produce high amount of urease (which accounts for up to 6% of total bacterial protein) as one of its adaptation methods to overcome stomach acidity. The mechanism involves the hydrolysis reaction of urea which results in ammonia and carbonic acid, and hence, neutralizes the strong acid in its environment, due to their buffering properties.

The arginase of *H. pylori* also plays an important role for acid protection in vitro, as the only pathway of arginine catabolism in *H. pylori* is that initiated by arginase [4,5]. In addition, another protection mechanism is involved, chemotaxis, *H. pylori* being able to detect the pH modifications in the gastric mucosa and move towards the less acidic region [6].

The *H. pylori* genome has been completely sequenced in 1980 being isolated from a patient suffering from gastritis [7]. It has a circular chromosome of 1,667,867 base pairs and 1590 predicted coding sequences [8]. The results suggest that the basic mechanisms of replication, cell division and secretion are similar to those of *E. coli* and *H. influenza* [8]. However, the size of its core genome (namely the number of genes present in isolates) varies depending on the number of analyzed strains and their geographical origin. Also, based on genome investigation, the virulence of *H. pylori* species has been related to their ability to produce a cytotoxin-associated protein (CagA) and active vacuolating cytotoxin (VacA) [9]. VacA is known to damage epithelial cells, disrupts tight junctions, and causes apoptosis, while CagA may cause inflammation and is a potentially carcinogen agent [10].

Actually, due to existing diversity between strains of *H. pylori*, the specific strain that infects a person can predict the outcome. On the other hand, the clinical aspects and the epidemiology of *H. pylori* infections have been drastically changing in the last decades, especially in developing countries. Despite the treatment regimens and international guidelines developed by specialists in microbiology, there is currently a noticeable failure, mainly due to *H. pylori* resistance to several antibiotics, in the context of global increase in antibiotic consumption in the general population [11,12]. For example, WHO admitted that clarithromycin-resistant strains of *H. pylori* are among the twelve priority pathogens for which novel antibiotics or alternatives are urgently required [11,13]. Many efforts have been made in order to understand the mechanisms of antibiotic resistance and hence, the sound understanding of its genetics and physiology attracted a huge interest form both fundamental and applied microbiology researchers, along with ecology, medical, and veterinary disciplines [14,15,16].

In the context of increasing rates of antibiotic-resistant *H. pylori* strains, the risk factors and prevalence on global population, the aim of our work is to highlight the main drawbacks of currently used treatment regimens against *H. pylori* and at the same time, to emphasize the huge potential of natural alternatives, plants extracts and new formulation design and strategies to combat this pathogen. Special attention is also given to nanotechnological formulations, with huge potential for tissue microenvironment-responsive treatment.

## 2. Prevalence and Risk Factors Relevant for Therapeutical Support

### 2.1. Prevalence of H. pylori Related to Different Geographic Areas and Global Population

The prevalence of *H. pylori* infections varies worldwide in terms of epidemiological, demographic and socioeconomic criteria. A systematic review with meta-analysis from 73 countries, in six continents, conducted by Zamani et al. for the period 1 January 2000–31 June 2017, revealed an overall prevalence of 44.3% worldwide and it is suggested that approximately 60.3% of the world population was infected in 2015 [17,18].

In terms of geographical distribution, Latin America and the Caribbean have shown the highest rate of infection in the world (59.3%), while at the opposite pole, in the Northern America, the prevalence was only 25.8%. While in North and South America, the pooled data presented a good homogeneity, large variations in country-based prevalence were noticed in Asia and Europe. For example, a high rate was observed in the population of Kazakhstan (79.5%) compared to those from Indonesia (10.0%), while in European countries, Serbia seems to have the highest prevalence (88.3%) in opposition to Belgium (11.0%) [19]. As expected, in terms of demographic and socio-economic criteria, in developing countries the rates were higher compared to the developed ones (50.8% vs. 34.7%), which can be related to the lifestyle and environmental parameters, fact agreed by another meta-analysis reported by K.Y. Hooi et al. [18].

Although the risk of exposure increases with the age, some studies suggest that children who became infected with *H. pylori*, were infected at very young age (<5 years), and approximately 85% have long asymptomatic periods and clinical manifestations are non-specific [20,21]. Another study from 2006 in Ireland concluded that most children become infected with *H. pylori* at a very young age, and the risk of infection declines rapidly after 5 years of age [20]. Obviously, children living in the most crowded conditions are at the greatest risk for *H. pylori* acquisition. An inverse correlation was noticed between the mother’s education and *H. pylori* positivity in children. On the other hand, it is generally accepted that breast-feeding plays a protective role against the acquisition of *H. pylori* infection [22,23]. However, in children, the treatment should be considered in a different approach than in adults, as the immune mechanisms are different.

In terms of epidemiology, many studies have proposed that acquisition of *H. pylori* occurs via common environmental sources such as drinking water [24,25], animals or animal products [26,27,28]. The most probable route of transmission seems to be the ingestion route [29].

Even if *H. pylori*-associated disorders usually regress or heal completely after successful treatment with antimicrobials, the available antimicrobial therapies for *H. pylori* infection have many shortcomings and side effects. A combined therapy is very often required, especially because of the development of antimicrobial resistance [30].

### 2.2. Risk Factors: Oncogenicity

*H. pylori* is extremely variable in prevalence, taking into consideration risk factors such as: age, gender, socio-economic status, geographic area, and diet and lifestyle. It is widely accepted that lower socio-economic status is linked to poor hygiene conditions and lack of sanitary education, thus leading to higher rates of *H. pylori* incidence. That is why the prevalence of *H. pylori* infection is higher in developing countries and lower in high-industrialized countries. Beside the socio-economic status, deficient sanitation, and crowded living conditions, which are well known risk factors, some studies suggested that smoking, use of nonsteroidal anti-inflammatory drugs, 0 blood group, high body mass index and family history of gastric disease are also considered risk factors in the acquisition and transmission of *H. pylori* [31,32,33]. Diet may be another risk factor for *H. pylori* infection. Several epidemiological studies have revealed that good dietary habits such as consumption of green tea, fruits, vegetables with high levels of carotenoids, folate, vitamin C and phytochemicals are protective against *H. pylori* infection and gastric cancer risk [34,35,36,37,38].

**Figure 1:**
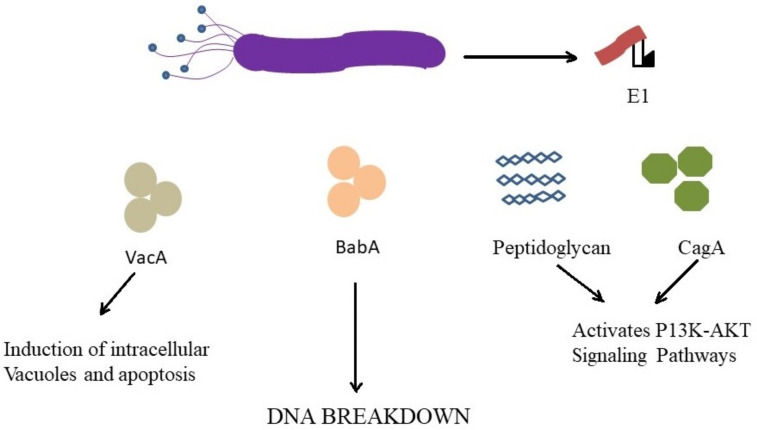
Carcinogenic effect of *H. pylori* through different mechanisms.

Salt and some transition metals such as iron, zinc, nickel, can alter *H. pylori* virulence and accelerate *H. pylori*-dependent disease progression [34]. *H. pylori* can also exert its carcinogenesis effect directly over its virulence factors such as cytotoxin-associated gene A (CagA) containing the Cag Pathogenicity Island (CagPAI), vacuolating cytotoxin A (VacA). The strains protecting CagA can cause more inflammatory responses (blood group antigen-binding adhesin A (BabA) and progression to gastric cancer. CagPAI encodes a bacterial type IV secretion system which secretes CagA and peptidoglycan into the epithelial cells. CagA and peptidoglycan induce multiple cellular signaling pathways such as MAPK cascade and phosphoinositide-3 kinase (PI3K-AKT) signaling pathways, respectively, as described in Figure 1**.** The explanation is based on the fact that the most important virulence factors expressed by *H. pylori*, VacA and CagA toxins, are transcriptionally regulated in by iron [39].

On the other hand, several studies have shown a correlation between zinc intake and *H. pylori*-dependent disease progression in the Asian population, which are well known related to CagA strains of *H. pylori* [34,40,41,42]. There are also differences between genders and age. A systematic review and meta-analysis published in 2017 showed that male gender is associated with a higher prevalence rate for *H. pylori* than the female gender, both in grown-ups and in children [43]. In a study from 2003 that included 307 children and 604 adults, *H. pylori* was diagnosed in 40 children and 308 adults [44]. There is also evidence that it plays a significant role in gastric cancer [45,46,47,48,49,50]. Gastric cancer is the number 5th most frequently diagnosed malignancy worldwide. It has a poor life expectancy despite medical treatment, being the 3rd cause of death due to cancers worldwide [45]. *H. pylori* has been recognized as a first-class risk factor for gastric malignancies by the International Agency of Cancer Research since 1994. Based on histological characteristics, gastric cancers can be classified in two major types: adenocarcinoma and lymphoma of gastric mucosal-associated lymphoid tissue (MALT). According to several scientific data, the second one is less common than adenocarcinoma (only 3% of all gastric tumors), while MALT-lymphoma is in 90% of cases caused by *H. pylori* infection [46,47]. Effective eradication of *H. pylori* has been proved to improve clinical outcome of patients suffering from gastric ulcers, preventing clinical relapses, lowering the risk of MALT-lymphoma incidence and inducing regression in diagnosed MALT-lymphomas [51,52,53]. Therefore, it is of crucial importance to evaluate the time frame in which *H. pylori* causes a carcinogenic effect, because beyond this time point, the intervention is significantly less effective. Taking into account the virulence factors and metabolic processes of *H. pylori* in developing gastric carcinogenesis, a complex approach should be consider including multiple measures and usage of appropriate antibiotics, eventually combined with natural biomedicines are necessary in order to eradicate this bacterium.

## 3. Current Treatment Regimens for *H. pylori* Infections

*H. pylori* seen as a bacterial infection, has been intensely studied, concerning a proper treatment. Although in vitro testing made it seem sensitive to a wide range of antimicrobials, in vivo only a few are useful [54]. Even so, there are more factors that must be taken into consideration such as: doses, formulations, frequency of dosing in relation to meals, treatment duration, use of anti-secretory drugs, antiacids, or probiotics [55]. The success of the therapy for many antimicrobial drugs is greatly diminished by special conditions such as acidic pH, formation of a biofilm but antibiotic resistance is the most important cause of failure of *H. pylori* eradication. The mechanisms underlying antimicrobial resistance are well known and they are also applicable for *H. pylori*. There are only a few antibiotics active against *H. pylori*: clarithromycin, amoxicillin, metronidazole, fluoroquinolone (levofloxacin, norfloxacin) tetracycline, rifabutin. Of concern is the increase in resistance to clarithromycin and metronidazole. It is currently accepted that *H. pylori* effective therapy requires two or more antimicrobials for a period of 14 days, along with an antisecretory drug to reduce gastric secretion.

The best treatment antibiotic option is established by drug-susceptibility testing which identify susceptible and resistant strains. There are many methods for this purpose, Kirby Bauer method, microdilution, E-test but all are time consuming and not available in all laboratories. Instead, a better option are molecular methods that detect specific changes in the *H. pylori* genome [56,57,58].

### 3.1. Clarithromycin Triple Therapy

International guidelines for treating infection with *H. pylori* are consistent with the use of triple therapy as the first-line treatment. Triple therapy consists of the administration of a proton pump inhibitor (PPI), clarithromycin, and amoxicillin for one-two weeks [59,60]. Currently, concomitant therapy (PPI, amoxicillin, clarithromycin, and a nitroimidazole administered concurrently) should be the preferred non-bismuth quadruple therapy, as it has shown to be the most effective to overcome antibiotic resistance according to the Maastricht V Convention [59]. The clarithromycin regimen is one of the first and most effective eradication therapies. The regimen has a high-eradication rate >95%, but the best results are obtained if the regimen is followed for 14 days [61,62]. For various reasons, sometimes is prescribed for 7 or 10 days, leading to lower rates of eradication mainly due to an increase in clarithromycin resistance [63]. Other regimens called second-line therapies have been proposed and applied [63]. These treatments consist of an association of PPI with two or three antibiotics: amoxicillin, clarithromycin, metronidazole. Lately, it was proven that rates of eradication go as far as 70%, which is considered very low for an infectious disease. Concomitant therapy is preferred over sequential one because it is easier to be administrated. There are controversies about superiority of concomitant therapy toward sequential therapy [64,65,66,67], but there is relevant evidence proving that there is no significant difference in the eradication rate between concomitant therapy and sequential therapy [68,69]. In case of resistance to both metronidazole and clarithromycin, the alternative candidates for third-line therapy are quinolones, tetracycline, rifabutin, and furazolidone, but the rate of resistance to metronidazole and quinolones used as second- or third-line regimens is more than 15% worldwide [70,71,72].

### 3.2. Bismuth Quadruple Therapy. Non-Bismuth-Based Quadruple Therapy

The bismuth quadruple therapy consists of administrating bismuth, proton-pump inhibitors, tetracycline, metronidazole or tinidazole for a period of 7–14 days. In the 19th century bismuth was widely used in gastroenterology. Since 1970, bismuth was used as colloidal bismuth subcitrate (De-Nol), as an ulcer medication. It has many pharmaceutical properties such as: bactericidal effect on *H. pylori*, binding to the ulcer base, inactivation of pepsin, binding of bile acids, stimulation of prostaglandin biosynthesis, suppression of leukotriene biosynthesis, stimulation of complexation with mucus, inhibition of various enzymes, binding of epithelial growth factor, stimulation of lateral epithelial growth [73]. The bismuth quadruple therapy has an excellent *H. pylori* eradication rate (more 80%) therefore is considered a second line therapy [74,75,76]. Bismuth was used as a medical agent ever since 1697 [76]. Its disadvantages are the inaccessibility to bismuth in some countries, the rising resistance to metronidazole and low adherence to the regimen [77]. A single capsule therapy containing bismuth, metronidazole, and tetracycline has been developed and administrated with large successful rates [78]. According to a meta-analysis, first- and second-line single-capsule therapy as described above achieved an eradication rate approaching 90% [79]. The non-bismuth-based quadruple therapy consists of adding metronidazole to the classic PPI, amoxicillin, and clarithromycin. It is efficient in clarithromycin resistant populations, but it has not been studied for metronidazole resistant populations. Eradication rates can go as high as 88%. It is safe to use, efficient, but it has gastro-intestinal side-effects [80,81].

### 3.3. Rifabutin-Based Triple Therapy

Rifabutin is a structurally similar to rifampicin use as anti-tuberculosis drug, mainly for atypical tuberculosis. The action of rifabutin consists in stopping protein synthesis by inhibition of the beta-subunit of the *Helicobacter* DNA-dependent RNA polymerase. This enzyme is encoded by RpoB. In case of mutation of RpoB results resistance to rifabutin [82]. The rifabutin-based triple therapy used as a third-line therapy in patients that failed to response to classic antibiotic treatments such as amoxicillin, clarithromycin, metronidazole, tetracycline, and levofloxacin. It consists of rifabutin, amoxicillin and a PPI taken for a period of 14 days. The ideal dosage is 150–300 mg rifabutin daily. The therapy with rifabutin has many advantages (fat-soluble, good absorption after oral intake, stable at a wide range of pH values), but the most important is low the rate of resistance in *H. pylori* because it is rarely used clinically [83]. Rifabutin-based regimens are important for rescue therapies, but the eradication rate is different reported and future related studies are needed. Overall, the efficacy rates of eradication are 73%. Its disadvantages include leucopenia and myelotoxicity [84,85].

### 3.4. Fluoroquinolone Therapy

The levofloxacin triple therapy is used because of increasing resistance to clarithromycin. The ideal dose is 2 mg × 500 mg of levofloxacin daily, with fewer side-effects than the bismuth quadruple therapy. It is used as a second-line rescue therapy, after first-line eradication treatments fail [86]. New generations of fluoroquinolones are levofloxacin, moxifloxacin, gatifloxacine, and pefloxacine. They have a broad-spectrum activity and are used in infections of the respiratory and urogenital tract. Because they are more frequently used, the bacterial resistance, including *H. pylori,* is rising [87]. Data containing other fluoroquinolones, other than levofloxacin, are rare. Moxifloxacin used with esomeprazole and tinidazole or moxifloxacin with esomeprazole and amoxicillin had eradication rates of about 88–90% in a study from 2005 [88,89]. A study from India showed an eradication success of up to 77% for a 2-week dual therapy with proton pump inhibitors and norfloxacin [90,91]. They are used as a second line therapy in countries where bismuth is not available or where the population has high rated of clarithromycin resistance.

### 3.5. Potassium-Competitive Acid Blockers (P-CABs)

Potassium-competitive acid blockers are a group of drugs that inhibit gastric secretion faster and with a longer action. They seem to be more potent than PPI because they do not require activation by stomach acid and their efficacy is not influenced by various subtypes of cytochromes P450. The P-CABs are bases that inhibits the H^+^ K^+^ ATPase enzyme, key enzyme in formation of HCL. In addition, the protonated form of this drug is less able to pass via cellular membranes than the nonionic molecules, so they concentrate into the canaliculi of parietal cells, the place where HCl is produced [92]. The efficacy for *H. pylori* eradication was assessed in more multicenter studies using P-CABs in association with antibiotics (triple therapy). The eradication rate was 94.4% for the first-line therapy and 97.1% for the second-line therapy. The conclusion was that P-CABs-triple therapy was safe and effective [79], while the eradication and the incidence of adverse events was 4.4% [93].

## 4. Antibiotic Resistance and Alternative Therapy

**Figure 2:**
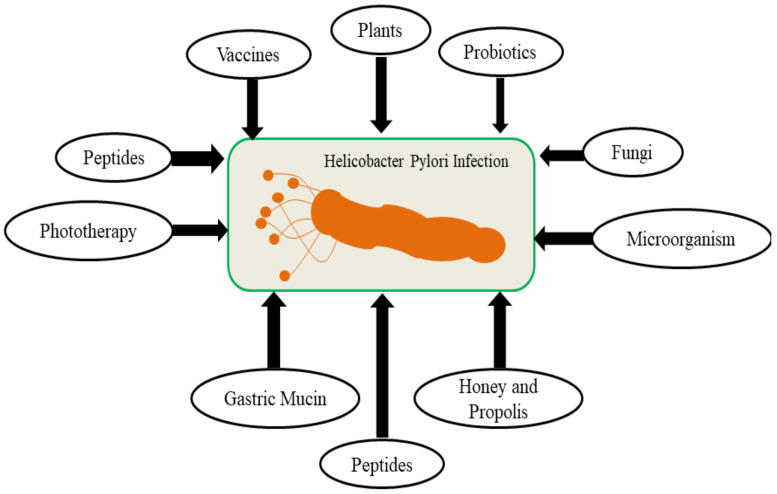
*H. pylori* infections and alternative treatment approaches.

The 1980 discovered *H. pylori* bacteria is nowadays considered as the etiologic agent for gastric ulcers and gastric cancer. Earlier, various drug regimens were used for the treatment and cure of such bacterial infections. However, now, the alarming situation of increasing resistance to antibiotics, moreover the high-cost therapy, resulted into the emergence of alternative approaches such as anti-inflammatory agents, anti-oxidants and most importantly, herbal plants and their extracts. These alternative approaches inhibit and modulate the release of inflammatory cytokines, reactive oxygen species and other inflammatory mediators which generate on the interaction of *H. pylori* with gastric mucosa and ultimately lead to atrophy, dysplasia, metaplasia and changes in the gastric gland. The herbal extracts have shown important roles such as anti-inflammatory, antioxidant, anti-cancer, and bactericidal against *H. pylori* [94,95]. There are alternative therapies for the treatment *H. pylori* infection as shown in Figure 2.

As a result, there is an increasing trend of herbal drug therapy in almost 80% of population, especially those who found modern antibiotic therapy not affordable, not accessible, and not accepted. Various plants and their extracts show promising results against *H. pylori* [94]. A selection of the most relevant natural extracts was performed by searching the full text information in databases of PubMed, Web of Science, Embase, and Google Scholar, with priority within the time interval 2005–present, as presented in Table 1.

In the next section, a more detailed discussion is provided, referring to propolis, curcumin, ginseng, and garlic extracts, as a valuable source of active ingredients against *H. pylori* mediated disease, due to their easy accessibility and affordability for population.

### 4.1. Role of Propolis in Treatment of H. pylori Mediated GIT Diseases

One of the important and beneficial natural treatments for *H. pylori* infection is propolis, produced by honeybee *Apis melifera*. Different studies conducted all over the world reported that caffeic acids, flavonoids, and phenolic esters are the main biologically active compounds in propolis [126,127]. Specifically named chemical compounds (to mention only a few) arechalcones-2,6-dihydroxy-4-methoxychalcone and 2,4,6-trihydroxy-4-methoxy-chalcone, benzoic acid derivatives such as salicylic acid, 4-methoxybenzoic acid, 4-hydroxybenzoic acid, procatechuic acid, gallic acid, etc., benzaldehyde derivatives such as vanillin and isovanillin, cinnamyl alcohol, cinnamic acid and its derivatives, terpene, sesquiterpene alcohol and their derivatives, aliphatic hydrocarbons, sterols and steroid hydrocarbons, sugars such as d-glucose, d-fructose and ribofuranose, amino acids, and some minerals such as Na, K, Mg, Zn, Cd, Ca, Ag, Ni, Fe. These mentioned compounds and their derivatives account for the pharmacological importance of propolis [128].

The bioactive phenolic compounds are present as free or alkyl and phenyl esters which are responsible for various antioxidant, antimicrobial, anticancer and anti-inflammatory properties of propolis. According to literature, 21 polyphenols were detected in propolis which account for the anti *H. pylori* activity [128,129,130,131]. The main compounds and their combination of chrysin, pinocembrin, galangin and caffeic acid phenethyl ester showed the bactericidal effect against *H. pylori*. The research studies conducted by Shapla et al. [128] have proven the scientific evidence in treatment of *H. pylori* infection and clearly justifies the protective role of propolis phenolic compounds in decreasing and neutralizing the host and bacteria’s physiology. Hence, they suggest propolis as an alternative treatment approach in *H. pylori* associated gastrointestinal diseases [128].

#### 4.1.1. Anti-Inflammatory Effect of Propolis

The caffeic acid phenethyl ester (CAPE) obtained from propolis also has anti-inflammatory properties. It shows immunosuppressive action on T cells of host and also inhibits cell activation cycle. It also shows inhibitory effect on other inflammatory mediators such as NFkB and transforming growth factor. In an in vitro study CAPE blocks the MAPK signaling and NFkB activation and ultimately decrease IL-8 production, because IL-8 production is an important pathological role in *H. pylori* induced gastritis. In addition to the inhibitory effect on mediators, it also shows its action on enzymes such as myeloperoxidase, and NADPH-oxidase, and this activity indirectly inhibits neutrophils and other inflammatory cells infiltration. The other important enzyme peptide deformylase in pathogenesis is also competitively inhibited by CAPE. The oxygen free radicals produced by Xanthine oxidase also cause tissue damage and inflammation which are inhibited by propolis extract [132,133].

#### 4.1.2. Antioxidant Effect of Propolis

Through research studies propolis is found to have higher antioxidant effects than green tea. It contains various polyphenols and flavonoids which show antioxidant properties in many ways such as hydrogen donors, reducing agents, chelating ions, etc. It protects the blood cells from oxidative stress induced by free radicals and maintain their cellular structures, rigidity, and their functions [126,133,134]. Hence, based on several evidence presented, the role and benefits of propolis are clearly against *H. pylori* infection. Therefore, it can be concluded that propolis may be used as an alternative therapeutic approach in the treatment and ailment of the gastrointestinal diseases such as ulcers and gastritis.

### 4.2. Role of Curcumin in Treatment of H. pylori Mediated GIT Diseases

Curcumin is a well-known polyphenolic bioactive yellow color pigment obtained from rhizomes of curcuma longa also known as turmeric. It is widely used as a spice and food coloring agent especially in India. Various research studies previously shown multiple health benefits such as antioxidant, anti-inflammatory, anti-cancer, and anti-microbial, with the approach of anti-angiogenesis for the prevention and treatment of certain diseases such as cancer, chronic inflammation, or atherosclerosis [135]. The invitro studies and preclinical trials also gave results on its effect on *H. pylori* and its induced diseases. From a chemical point of view, curcumin exists as a keto-enol tautomer, where the enol isomer is more stable than keto form, in both solid and liquid state. The yellow color is found at acidic pH, while basic pH shows red color of curcumin. The polyphenolic chemical compounds possess two aromatic rings with two unsaturated carbonyl groups. This molecule is stable due to the central hydroxyl group (-OH) which is found to be the crucial functional group for antioxidant and other biological activities [136].

#### 4.2.1. Antioxidant Effect of Curcumin

The curcuminoids in alcoholic extract reported to act as scavengers for oxygen free radicals. They interact with reactive oxygen intermediates and protect the cells from damage. Curcumin is found to be more stable at acidic pH in GIT. Oxidative stress and damage play a vital role in pathogenesis of *H. pylori* induced diseases. So, it has shown to be a potent oxidative scavenger to treat oxidative induced diseases [137].

#### 4.2.2. Anti-Inflammatory Effect of Curcumin

The anti-inflammatory effect of curcumin is already well known. Curcumin inhibits carrageenan- induced paw edema in mice in various inflammatory diseases such as ulcerative colitis in preclinical trials. The mechanism behind the anti-inflammatory effect is inhibition of nitric oxide synthase, cyclooxygenase-2, and inflammatory mediators such as cytokines (IL-1, IL-6, IL-8, IL-12), tumor necrosis factor-α etc. It also inhibits prostaglandins along with lipoxygenase activity [138].

#### 4.2.3. Anti-Carcinogenic Effect of Curcumin

Curcumin has also anti-cancer property and plays an important role in treatment of gastric and other types of cancer. The inflammation and malignancy are correlated. The inhibition of I-Kβ kinase, and various transcription factors reduces the effect of inflammatory mediator NF-Kβ and synthesis of cytokines, respectively, which is involved in pathogenesis of cancer. Along with gastric cancer treatment it is also used in oral, esophageal, duodenal, and colon cancer [139].

#### 4.2.4. Anti-Microbial Effect of Curcumin

Curcumin inhibits the growth of various bacteria’s and parasites including *H. pylori*, *Bacillus subtilis*, *Plasmodium falciparum*, *Leishmania*, etc. Curcumin, the Indian gold has all the properties from being an antioxidant to a beneficial anti-inflammatory agent, that is why it is used in treatment of *H. pylori* bacterial infection. The invitro studies by Marathe et al., gave results on inhibition of growth of 19 different strains of *H. pylori* [140]. Using a mouse model, Vetvicka et al. [141] studied the effects of curcumin on lipid peroxide level, myeloperoxidase and urease activity, along with levels of anti- *H. pylori* antibodies, biofilm formation, and levels of IFN-γ and IL-4 in serum. They found significantly increased IL-4 and decreased IFN-γ serum levels, while the levels of LPO (an oxidative damage index) were decreased by supplementation with curcumin. The results of this study also support the hypothesis that curcumin has strong immunostimulating properties.

Hence, curcumin can be used either alone or in combination with other antibiotics to cure and treat *H. pylori* induced gastric diseases due to its multiple benefits.

### 4.3. Role of Ginseng in Treatment of H. pylori Mediated GIT Diseases

Since centuries, Ginseng, obtained from the roots of *Panax ginseng* is a widely used herb in Asian folk medicine. The antioxidant, anti-inflammatory, anti-microbial, and anti-cancer properties of ginseng have already been proved in many pharmacological and clinical studies [142,143,144]. Ginseng mainly contains saponins called ginsenosides, which are secondary metabolites, responsible for pharmacological activity. Actually, the structural diversity of ginsenosides contributes to the multiple pharmacological benefits of ginseng on cancer and diabetes treatment, inflammation, stress, immune, cardiovascular system, and central nervous system disease.

The acidic polysaccharide from ginseng helps to dislodge the *H. pylori* from human gastric epithelial cells and Ginsenoside Rb1, an important primary Ginsenoside improves inflammation by inhibiting pro-inflammatory mediators mainly cytokines and TNF-α.

Ginseng inhibits DNA mutagenesis, pathogen mediated hemagglutination, improves immune–modulatory functions and exerts bactericidal effect and protects the host from pathogen induce inflammation. The lactic acid present in fermented ginseng extract also exhibits potent anti- *H. pylori* activity [144].

Another research study shows the beneficial effect of red ginseng as it exerts protective function against carcinogenesis in gastric mucosal cells [143]. Red ginseng inhibits ERKI/2 signaling which on further inhibits DNA damage and apoptosis induced by *H. pylori*. It also inhibits synthesis of pro-inflammatory mediators especially IL-1β, iNOS, MOP activity. Panaxytriol shows effective inhibition of *H. pylori* growth and Ginsenoside Rh1 and protopanaxatriols inhibits the terminal stage H^+^/K^+^ ATPase of stomach acid secretion [145,146].

White ginseng extract has also shown antimicrobial, anticancer, and anti-inflammatory effects. Through disk diffusion assay study, it has been concluded that it inhibits the bacterial growth effectively. Additionally, it exerts cytotoxic effects and used in treatment of lung cancer, endometrial and uterine carcinoma. Moreover, the anti-inflammatory effect is shown by inhibiting nitric oxide production and activity [144,145]. In a very recent paper, [147] it was demonstrated the inhibitory effect of Korean red ginseng extract on DNA damage response and apoptosis in *Helicobacter pylori*-infected gastric epithelial cells, by increasing apoptotic indices and ROS levels. The authors suggests that supplementation with Korean red ginseng is beneficial for preventing the oxidative stress-mediated gastric disorders associated with *H. pylori* infection.

Therefore, it can be concluded that ginseng may be used as an alternative or complementary therapeutic approach in the treatment of *H. pylori* induced infections.

### 4.4. Role of Garlic in Treatment of H. pylori Mediated GIT Diseases

Currently, the principal medicinal uses of garlic are to prevent and treat cardiovascular disease by lowering blood pressure and cholesterol, as an antimicrobial agent, or as a preventive agent against different types of cancer. The antimicrobial activity of allicin, a diallyl thiosulfinate compound, was identified in garlic by Cavallito and Bailey in 1944 [148]. The garlic extract was found to be effective against *H. pylori* infection, as thiosulfinates (allicin) play an important role in the antibiotic activity of garlic [96]. Based on a systematic review and meta-analysis, Xiao-Bei Si et al. [98] highlighted the output of clinical trials that included allicin as an add-on treatment to triple therapy or bismuth containing quadruple therapy for *H. pylori* infection. The results of this meta-analysis revealed that allicin treated groups showed an eradication rate of ~93.81%. Additionally, higher healing rates and total remission rates of peptic ulcers were evidenced, and different scenarios for the mechanisms involved in ulcer response were proposed: (i) the activation of NF-Kβ was inhibited by allicin, which further inhibits the production of TNF-α, leading to anti-inflammatory effects; (ii) garlic extracts possess a protective role against oxidative stress in gastric tissue, which cause mucosal injury and ulcer development; (iii) garlic extracts favors NO synthesis, increasing the activity of nitric oxide synthase with crucial role in endothelial function [98].

The Feldberg in vitro research study has elucidated the mechanism behind the antimicrobial activity [144]. Allicin mainly inhibits the mRNA degradation as well as RNA, DNA and protein synthesis in the cells, where RNA polymerase is the major target of allicin. The major garlic materials available are garlic powder and garlic oil. Garlic powder is prepared by slicing, drying, and pulverization of garlic cloves, whereas the oil is prepared by heating the cloves and collection of its vapor. These commercial formulations are widely used for its antimicrobial activity against *H. pylori* [97]. Chemopreventive activity of garlic was also demonstrated either as fresh extract, garlic oil, or organosulfur compounds derived from garlic [149,150,151]. Garlic is also rich in antioxidants which destroys free radicals that can damage the cell membrane of epithelial cells, inducing gastric cancer. The antioxidant activity of garlic is mainly due to the organosulfur compounds derivatives which inhibit the pathological mechanisms involved in cancer, including free radicals formation, mutagenesis, angiogenesis, DNA formation, and cell proliferation. The cell proliferation is inhibited by garlic, mainly acting on G2/M phase of cell cycle [96,152].

With the evidence provided by the studies, it is clear that garlic serves as beneficial alternative therapy to antibiotics against different gastrointestinal tract diseases induced by *H. pylori*.

## 5. Nanotechnology-Based Approach against *H. pylori* Infections

Metallic NPs such as silver, gold, zinc or iron have been previously reported to possess the ability of killing a wide range of bacteria including *H. pylori* [153,154] by well-known underlying mechanisms involving oxidative stress, metal ion release and nonoxidative stress. A very low NPs concentration is necessary for bactericidal effect, and hence, it is difficult for the bacteria to develop resistance. Among different metallic NPs, AgNPs are convenient, especially the biologically derived ones, as the preparation methods demonstrated a controlled particle size, shape, and mono-dispersity, while reducing time of preparation, in the context of environmentally friendly approaches.

### 5.1. Role of Biologically Synthesized Silver Nanoparticles

Biosynthesis of silver nanoparticles using different plants extracts, along with other biological sources such as bacteria, fungi, algae, emerges as a good alternative production, using nontoxic, clean and eco-friendly approach, allowing to modulate its physicochemical parameters. The concentration of plant extract and the substrate, reaction temperature and pH strongly affect size, shape and stability of silver nanoparticles. The number of publications dealing with AgNP synthesis with the assistance of various plant extracts (leaves, stems, roots, etc.) is huge, but recently, several papers aiming to highlight the importance of biosynthesized AgNPs, demonstrated that the main advantage of AgNPs synthesized from plant extract is that they are more stable compared to microorganisms synthesized nanoparticles [155,156]. The study conducted by Gurunathan et al. [157], AgNPs were biologically synthesized using leaf extract of Artemisia princeps as a bio-reductant and evaluated against *H. pylori*. The average size distribution of the synthesized particle was between 10–40 nm. The main results of this study demonstrated a dose-dependent decrease in cell viability and biofilm of *H. pylori* formation, potentiation of reactive oxygen species generation and DNA fragmentation in *H. pylori.* Moreover, cytotoxic effect against human carcinoma cells was evidenced [157]. According to literature [158], the mechanism of AgNPs action on cells involve several steps: adhesion on the surface of the bacterial cell wall (especially observed for Gram-negative bacteria, knowing to have a thicker cell wall), penetration into the cell and disruption of intracellular organelles and biomolecules, induction of oxidative stress, and modulation of signal transduction pathways. It is generally accepted that the bactericidal properties of AgNPs are associated with a disruption of RNA transcription, purines, pyrimidines, and fatty acids of bacteria, while upon interaction of AgNPs with cell DNA, the replication can break [158].

Muhammad Amin et al. [159] performed the green synthesis of silver nanoparticles using methanol extract from the plant *Solanum xanthocarpum* and analyzed its anti *H. pylori* activity in vitro, by comparing with four standard drugs: amoxicillin, clarithromycin, metronidazole, and tetracycline.

The results of this study indicated a stronger anti- *H. pylori* activity of AgNPs compared to AgNO_3_ and metronidazole, showing almost similar results to tetracycline, but less potent than amoxicillin and clarithromycin. Moreover, a significant inhibition was noticed in the *H. pylori* urease inhibitory assay. It was suggested that biological silver nanoparticles act as a potent therapeutic compound with anticancerous and antimicrobial properties, opening a new area of research for large medical applications of silver nanoparticles, including eradication of the *H. pylori* mediated gastric diseases [159]. Besides silver, other metallic nanoparticles such as bismuth, zinc, and gold were proved to be effective in the treatment of *H. pylori*, as reviewed by Safarov et al. [160]. Although there are enough evidences of the antimicrobial effect of metallic nanoparticles, there are also limitations, as most of them tend to accumulate in kidneys and liver, leading to cytotoxicity.

### 5.2. Other Nanoparticles Formulations for H. pylori Mediated GIT

#### 5.2.1. Polymeric Nanoparticles Encapsulating Drugs and Other Therapeutic Agents

Polymer-based nanocarriers are advantageous formulations when it comes to gastric delivery as they exhibited very good stability and loading capacity. The drug or therapeutic molecules can be directly encapsulated with polymeric nanoparticles or covalently conjugated to their surface. The role of nanocarriers is to protect the bioactive compounds in harsh gastric environments, to enhance the internalization by microbes and eradicate them in multiple pathways.

PLGA (poly lactic- co- glycolic acid) are good candidates because due low toxicity and possibility to encapsulate low water-soluble drugs such as clarithromycin, in order to reach the target region [161,162]. PLGA nanoparticles loaded with clarithromycin, in size of about 360 nm, have shown MIC values in the range 0.003 µg/mL to 0.05 µg/mL, which were significantly lower than non-encapsulated drug solution, as demonstrated for clinical five *H. pylori* strains [162]. Gliadin, a biodegradable, biocompatible, and natural origin biopolymer, is known to adhere to mucosa membranes. Thus, its mucoadhesive properties is advantageous for the development of nanoparticulate delivery systems against *H. pylori*, as demonstrated by Ramteke S. et al. [163]. When using omeprazole and clarithromycin together, in a gliadin nanoformulation, the anti- *H. pylori* effect was found to be over 80%. Clarithromycin was also encapsulated into ethylcellulose nanoparticles and its activity against *H. pylori* was investigated by Pan-In, P. et al. [164] in animal model (rats).

The encapsulated clarithromycin was more effective than the free clarithromycin formulation, when administrated in rats. Actually, the initial reason for the antibiotic encapsulation was mainly due to its susceptibility to the highly acidified environment in the stomach.

Chitosan is another non-toxic polysaccharide, cationic in nature, with mucoadhesive properties, and widely used in the form of nanoparticles for gastric delivery of different bioactive compounds. It is composed of D-glucosamine and N-acetyl-d-glucosamine units, being insoluble at neutral pH, while rapidly ionizes and dissolves under the acidity of stomach [165]. The potential utility of chitosan micro and nanoparticles in the treatment of gastric infection was evaluated by Gonçalves et al. [166]. Enhanced effect of encapsulating amoxicillin into chitosan nanoparticles to treat *H. pylori* was demonstrated in vivo using Wistar rats, highlighting that *H. pylori* inhibition was not influenced by particle size [166]. Alginate, heparin, and poly-g-glutamic acid are anionic polymers commonly used for preparation of chitosan-based polyelectrolyte complexes, in the form of micro- or nanoparticles, and have been developed for encapsulation of antibiotics for *H. pylori* treatment [167,168]. According to literature, the novel strategy using targeted treatments based on chitosan micro/nanoparticles, specific to *H. pylori*, may include chemically modified (functionalized) particles with targeting compounds that bind *H. pylori* urease, flagella, adhesins, aiming to inhibit, kill or remove the bacteria, while avoid the destruction of the normal gut microbiome of the patients.

#### 5.2.2. Liposomal Formulations against *H. pylori*

Liposomes are also widely studied drug-delivery systems for antibiotic delivery, consisting of spherically assembled phospholipid bilayers, encapsulating various antibiotics, including metronidazole, ampicillin, amoxicillin, and doxycycline against to *H. pylori* [169]. The encapsulation efficiency of these drugs was found to be relatively low, and the in vivo effect of these formulations against *H. pylori* has not been verified. Fatty acids such as stearic acid, oleic acid and linoleic acid have been encapsulated into liposomes and evaluated against the resistant strains of *H.* *pylori* [170]. The hydrodynamic diameter of these formulations was around 86 nm. Both the liposomal formulation and free linoleic acid were effective in killing spiral and coccoid forms of the bacteria. The results demonstrated that linoleic acid possess the most relevant bactericidal effect thanks to its increased permeability, being capable of completely eliminate eliminating *H. pylori* within 5 min at a concentration of 400 μg/mL [170]. Because of its amphiphilic nature, linoleic acid can be readily loaded into liposomes and subsequently fuse with bacteria for antibacterial activity. Moreover, in vivo tests (mice animal model) confirmed that linoleic acid-based liposomal formulation was efficient in killing *H. pylori* and reducing bacterial load in the mouse stomach [171]. Additionally, the treatment was efficient in reducing the levels of proinflammatory cytokines including interleukin 1β, interleukin 6, and tumor necrosis factor alpha.

Other liposomal formulations were prepared using incorporation of docosahexaenoic acid into nanostructured lipid carriers, which resulted in increased bactericidal efficacy at much lower concentration compared to free docosahexaenoic acid. The suggested mechanism was related to the fragmentation of *H. pylori* cell membrane upon interaction with nanoliposomes, and leakage of cytoplasmic content, as revealed by Transmission Electron Microscope images, acting very rapidly in a dose-dependent manner [172]. An interesting approach used an engineered liposomal formulation based on gold/chitosan nanoparticles adsorbed on the liposomes surface, which demonstrated a smart “on-demand” antibiotic delivery in a controlled manner: the more enzymes or bacteria present at the infection site, the more antibiotic molecules released in situ [173]. Such on-site release of antibiotics enables localize and rapid killing of *H. pylori*, and minimizes side effects.

## 6. Conclusions

*H. pylori* is responsible for a chronic, transmissible, infectious disease and the increasing prevalence of antibiotic resistance has complicated the therapy. All therapies should assume the possibility of antimicrobial drug resistance. As an infectious disease, therapy should be based on drug susceptibility testing, but it is not always available. Since its classification as a group 1 carcinogenic by International Agency for Research on Cancer, substantial effort has been allocated in the investigation of therapeutic alternatives beyond antibiotics. Clarithromycin triple therapies now typically produce less than 80% cure rates and thus is no longer acceptable. Bismuth and non-bismuth-based quadruple therapy are inaccessible in some countries, and often are accompanied by gastro-intestinal side-effects. The rate of eradication in the case of rifabutin-based triple therapy is about 73%, but its disadvantages include leucopenia and myelotoxicity, while fluoroquinolone therapy, as a second line therapy in countries where bismuth is not available or where the population has high rated of clarithromycin resistance, may offer eradication success of up to 77%. The P-CABs triple therapy is probably the most safe and effective against *H. pylori*, but the treatment may cause individual variability regarding the effect. Selection of appropriate treatment should be made taking into account the molecular mechanisms of pathogenesis and virulence factors engaged in the development of gastric disease, while keeping in mind that successful antibacterial therapies depend on susceptibility to given drugs, doses, formulations, use of adjuvants, treatment duration, and reinfection rates. In this respect, the use of natural compounds in *H. pylori* treatment has gained huge popularity due to their low side effects and low toxicity, as encouraging results from in vitro and in vivo studies were reported. This work offers an extensively referenced approach on natural medicines against *H. pylori*, including the significance of nanotechnology in developing new strategies for treatment of *H. pylori* infection.

## Figures and Tables

**Table 1:** Plants and their formulations as active agents against *H. pylori*.

Name/Family	Formulation	Main Outcomes In Vitro/In Vivo	Reference
*Allium sativum*/ Amaryllidaceae	Garlic oil and powder	In vitro (standard and clinical isolates)—significant loss of viability, inactivation irrespective of strain, depending on allicin concentration In vivo *(clinical trials)*-improves eradication rates, healing rates of ulcers, remission of peptic ulcers, synergism with conventional therapy	[96,97,98]
*Bridelia micrantha*/ Euphorbiaceous	Stem bark extract	In vitro (standard strains and clinical isolates)—killing rate depending on concentration and time (agar-well diffusion and MIC method) Antioxidant, anti-inflammatory properties	[99,100,101]
*Camellia sinensis*/ Theaceae	Young shoot extract	In vivo (animal model)—prevents gastritis, limits the localization of bacteria and VacA to the surface of the gastric epithelium. In vivo (clinical trial)—suppression of gastritis and prevalence of *H. pylori* in a dose-dependent manner. In vitro—inhibits the production and function of the urease enzyme.	[102,103,104]
*Daucus carota*/ Apiaceae	Essential oil of carrot seed	In vitro and in vivo *(animal model)*—co-administration with pantoprazole exhibits strong anti-*H. pylori* activity	[105,106]
*Eugenia caryophillus*/ Myrtaceae	Extracts of flowers	In vitro (standard strains and clinical isolates)—decrease viability of *H. pylori*, irrespective of the strain, at acidic pH, effective in gastritis	[107,108]
*Geum iranicum*/ Rosaceae	Aqueous extract of roots	In vitro- effective against clinical isolates of *H. pylori* resistant to metronidazole	[109]
*Hancornia speciosa*/ Apocyanaceae	Bark’s hydroalcoholic extract	In vivo (rodent model)- anti-*H. pylori* effect by increasing of gastric mucus formation and antioxidant properties of polymeric pro-anthocyanidins Anti-inflammatory, antibacterial, and anticancer properties.	[110,111]
*Myrtus communis*/ Myrtaceae	Essential oil extract	In vitro—effective against clinically isolated strains resistant to nitroimidazoles and clarithromycin. Antimicrobial, antidiarrheal, anticancer, antioxidant, antiulcer, anti-inflammatory and antidiabetic activity	[112,113]
*Malus domestica*/ Rosaceae	Apple peel extract	In vitro—prevents vacuolation in HeLa cells, antiadhesive effect against *H. pylori.* In vivo (short-term infection model mice)—inhibitory effect on *H. pylori* attachment, anti-inflammatory effect on *H. pylori*-associated gastritis, reversible inhibitory effect	[114,115]
*Olea europaea*/ Oleaceae	Olive leaf extract	In vitro (clinically isolated meticillin resistant strains)—regulates the composition of the gastric flora, selectively reduce levels of *H. pylori* Antiulcer, antioxidant and antimicrobial effects	[116,117]
*Prunus dulcis*/ Rosaceae	Polyphenol extract	In vitro (standard strains and clinical isolates)—effective against *H. pylori* with different virulence irrespective of the *cagA* and *vacA* status.	[118,119]
*Rumex aquaticus*/ Polygonaceae	Plant extract	In vitro (human adenocarcinoma gastric cells)—cytotoxic effects against AGS cells, inhibit the production of proinflammatory cytokines, antioxidant	[120,121]
*Stachys setifera*/ Lamiaceae	Aerial part extract	In vitro (isolates)—strong inhibitory effect	[101,122]
*Zingiber officinale*/ Zingiberaceae	Rhizome extract	In vitro—potent inhibitors of proton potassium ATPase activity and *H. pylori* growth. In vivo (pilot study)—significant *H. pylori* eradication rate in dyspeptic patients, additive effect along with antibiotics	[123,124,125]
Propolis/ *Apis melifera*	Ethanolic extracts	In vitro—anti-*H. pylori* and anti-urease activities, 92.1% inhibition rate, synergism with clarithromycin or metronidazole	[126,127,128,129,130,131,132,133,134]
*Curcuma longa*/ Zingiberaceae	Rhizome extract, polyphenolicrich extract of the root	In vitro (clinical isolates)—stabilizes the vacuolar membrane and prevents the escape of cytosolic pathogens, suppressor of the type 1 immune response. In vivo (mouse model)—increase IL-4 serum levels and IgG, decrease IFN-γ levels	[135,136,137,138,139,140,141]
Ginseng/ *Panax ginseng Meyer*	Korean Red Ginseng extract	In vitro (infected gastric epithelial cells)—reduces reactive oxygen species and prevents cell death In vivo (animal model Mongolian gerbils)—anti-inflammatory effect on *H. pylori*-induced gastric inflammation	[142,143,144,145,146,147]
